# The First Christmas at Dagenham Sanatorium

**Published:** 1914-01-03

**Authors:** 


### The First Christmas at Dagenham Sanatorium.

Christmas will long be remembered by the patients at the Sanatorium, Dagenham, as a day which will stand out clear and distinct from the everyday life at this institution. The festivities were able to be carried out in a bountiful manner, which was brought about mainly through the medium of the energetic sister-in-charge, Miss E. Jones, ably backed up by the staff. It was indeed a happy sight to see the radiant faces of some fifty of the workers of the country—who have been stricken with the terrible disease for which they are undergoing treatment to bring them back to health—as they trooped into the large and airy wards and sat down at their respective tables covered with spotless cloths and laden with all the appetising features of a right-down good Old English Yule feast. Roast poultry, sausages, ham, vegetables in abundance, and plum-puddings were there awaiting the onslaught which was shortly to be made in real earnest. In addition to this were apples, oranges, and all kinds of dessert, not forgetting the fun-bestowing bon-bon, which, after the feast, was much in evidence, and paper caps and all kinds of headgear, unique and grotesque in hue and design, were the order of the day.

The decorations were also of a most effective and pleasing character in the recreation rooms, while various designs and mottoes were pinned upon the walls, relieved by strings of gay paper and evergreens. The mottoes included several well-known catch phrases, among them being the following:—"Good old Lloyd George," "Say 99," "Don't Koff," "Are you putting on weight?" "Success to the nursing staff," etc. Hanging in the centre of the room was a huge teddy-bear—"The T.B. we prefer." After dinner the patients gathered round the fires in the recreation rooms to gossip and play games, greetings and seasonable jokes went round, and now and then a more or less melodious voice sang a Christmas carol. Every patient received a beautiful present by dipping into the large "lucky tub."

In the evening the whole of the patients, as well as the staff, joined in a whist-drive, for which prizes were given to the winners. During the interval music and singing were given by the staff and patients. Hearty cheers were given for the staff, and the singing of the National Anthem brought a thoroughly happy and enjoyable function to a close.

